# The complete chloroplast genome and phylogenetic analysis of *Salvia oxyphora* Briq. 1896 (Nepetoideae, Lamiaceae)

**DOI:** 10.1080/23802359.2022.2098860

**Published:** 2022-07-22

**Authors:** Yifei Pei, Guiping Zhao, Xiwen Li, Dade Yu, Ning Cui

**Affiliations:** a Institute of Chinese Materia Medica, China Academy of Chinese Medical Sciences, Beijing, China; b Central Laboratory, Shandong Academy of Chinese Medicine, Ji’nan, China

**Keywords:** *Salvia oxyphora*, complete chloroplast genome, phylogenetic analysis

## Abstract

*Salvia oxyphora* Briq. 1896 is a perennial herb in the family Lamiaceae native to Central Bolivia. In this study, the chloroplast genome of *S. oxyphora* was sequenced using the Illumina platform and was assembled for the first time. The complete plastid genome of *S. oxyphora* was 151,014 bp in length including a large single-copy (LSC) region of 82,293 bp, a small single-copy (SSC) region of 17,531 bp, and a pair of inverted repeats (IR) regions of 25,595 bp. The total GC content of this genome was 38.04%, and that of LSC, SSC and IR regions was 36.21%, 31.80% and 43.13%, respectively. A total of 114 unique genes of this genome have been annotated, including 80 protein-coding genes, 30 transfer RNA genes, and four ribosomal RNA genes. The maximum likelihood phylogenetic tree was constructed with 51 complete chloroplast genomes, illustrating the close relationship of *S. oxyphora* to the Brazil’s native medicinal species *S. splendens*. The chloroplast genome of *S. oxyphora* provides a foundation for further studies on the adaptive evolution and genetic diversity of the genus *Salvia*.

*Salvia* is one of the large genera in Lamiaceae family, containing approximately 1,000 species, and is well known for its medicinal and ornamental values (Li et al. 2013; Cui et al. 2020). The genus *Salvia* is extensively widespread in tropical and temperate areas, with three centers of distribution in the world: Central and South America (CASA), Central Asia/Mediterranean (CAM) and East Asia (EA) (Li et al. 2013). Because of the large number of species in this genus and their wide distribution, *Salvia* is regarded as an important genus for interspecific variations and adaptive evolutionary research (Walker and Sytsma 2007; Ha et al. 2018; Hu et al. 2020). The chloroplast genome is a versatile tool for studies on evolution and phylogenetics of plants because of its highly conserved sequence and structure (Daniell et al. 2016). The chloroplast genomes of an increasing number of species have been sequenced, assembled and published in recent years, benefiting from the fast development of high-throughput sequencing technologies. However, only nearly 50 *Salvia* chloroplast genomes have been published so far. Compared to the large numbers of species in *Salvia*, chloroplast genomes of many more species in this genus are still required to understand their interspecific variations and evolution. In addition, among the three distribution centers of *Salvia*, CASA contains about half of all species (approximately 500 species), but the number of the published chloroplast genomes in this region (approximately 10 species) is far smaller than those in the other two regions (Li et al. 2013). Therefore, the chloroplast genomes of species in *Salvia*, especially of those distributed in the CASA region, are urgently needed for a better understanding of *Salvia*’s genetic information. *Salvia oxyphora* Briq. 1896 is a perennial herbaceous plant with a tropical appearance that originated from central Bolivia (Wester et al. 2020). It grows between 1 and 1.5 meters tall, is seedless, and is often propagated asexually using artificial cuttings (Wood and Ray 2012). It has attractive glossy green foliage and large striking cerise pink flowers with a long flowering time from summer to autumn (Wood and Ray 2012). *S. oxyphora* has great ornamental value, but its research in genetics and evolution is still extremely rare. In this study, we reported the chloroplast genome of *S. oxyphora* for the first time to understand the evolutionary position of this species.

The fresh leaves were collected from the plant in La Paz Botanical Garden, La Paz, Bolivia (Location: 16°32′ S, 68°04′ W) which was identified as *S. oxyphora* by Ning Cui. The specimen and DNA were deposited at herbarium of the Institute of Chinese Materia Medica, China Academy of Chinese Medicinal Sciences, Beijing, China (Xiwen Li, xwli@icmm.ac.cn) under the voucher number SZ20190901. The total genomic DNA was extracted using the modified Cetyltrimethylammonium Bromide (CTAB) method (Doyle and Doyle 1987). With the aid of Illumina Hiseq 1500 platform (Illumina Inc., USA), we constructed the genomic library for 150 bp paired-end sequencing. The complete chloroplast genome of *S. miltiorrhiza* (NC020431) was used as the reference genome for extracting chloroplast genome reads (Qian et al. 2013). The complete chloroplast genome was assembled by SOAPdenovo (version 2.04) (Luo et al. 2012). The complete chloroplast genome of *S. oxyphora* (accession number MT156374) was submitted to GenBank after being annotated by Plann (Huang and Cronk 2015). The complete chloroplast genome of *S. oxyphora* was 151,014 bp in length including a large single-copy (LSC) region of 82,293 bp, a small single-copy (SSC) region of 17,531 bp, and a pair of inverted repeats (IR) regions of 25,595 bp. Besides, 114 unique genes were identified, including 80 protein-coding genes, 30 transfer RNA genes, and four ribosomal RNA genes. The total GC content of this genome was 38.04%, with 36.21%, 31.80% and 43.13% in the LSC, SSC and IR regions, respectively.

The chloroplast genome sequences of 47 *Salvia* species were downloaded from GenBank for phylogenetic analysis, together with those of *Mentha longifolia*, *M. spicata* as well as *Glechoma longituba* used as an outgroup. After alignment with MAFFT (version 7.310) (Rozewicki et al. 2019), the maximum likelihood (ML) tree was conducted using RAxML (version 8.2.12) (Stamatakis 2014) with the GTRGAMMA model and 1,000 bootstrap replicates. It was clear from the phylogenetic tree that the majority of nodes were supported by high bootstrap values (Figure 1). All *Salvia* species were clustered together in one branch, suggesting that the evolutionary classification of *Salvia* species was similar. The ML tree showed that *S. oxyphora* was closely related to *S. splendens*, one medicinal plant originated from Brazil. The chloroplast genome of *S. oxyphora* provides a foundation for further studies on the adaptive evolution and genetic diversity of the genus *Salvia*.

**Figure 1.**
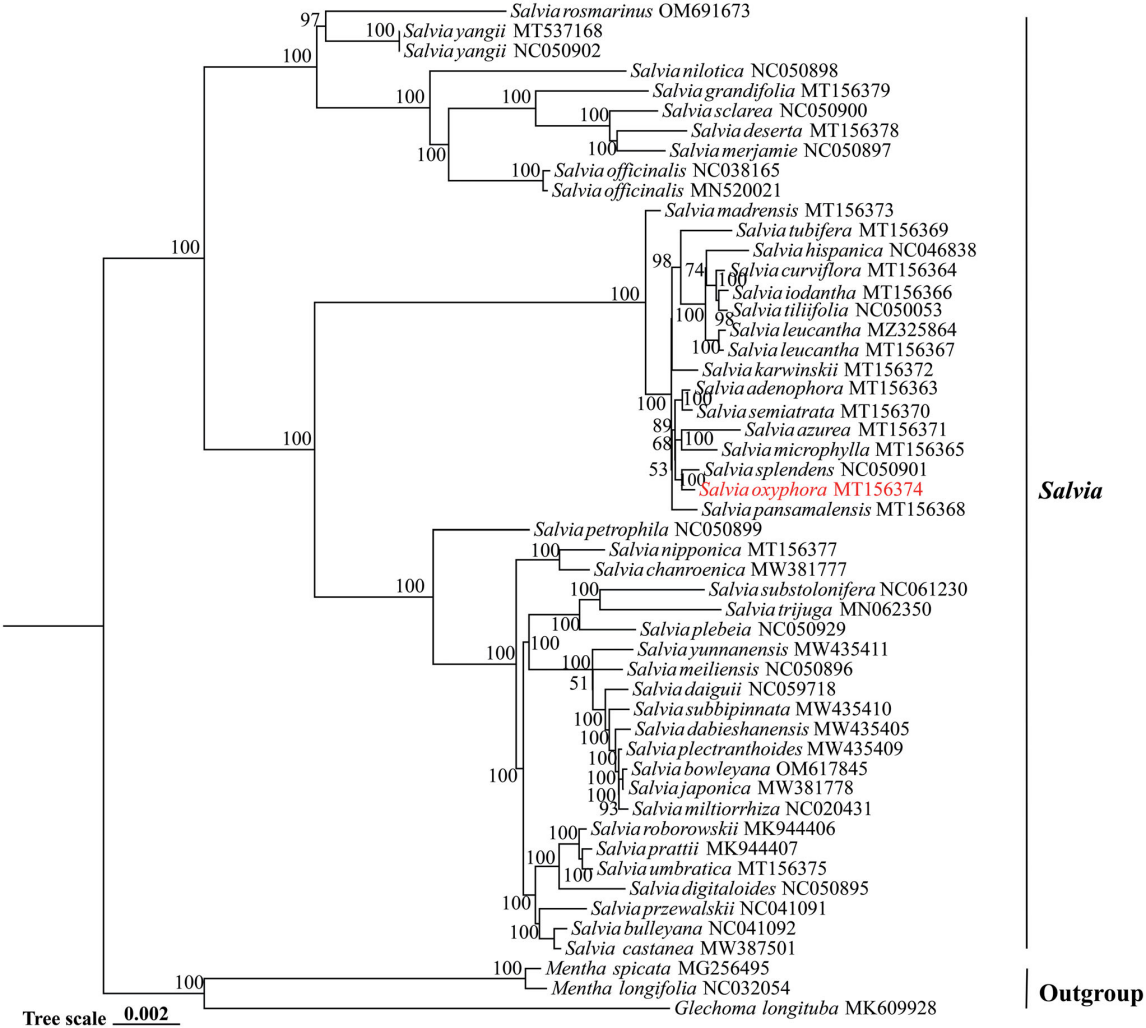
The maximum-likelihood (ML) tree was constructed based on complete chloroplast genome sequences of 48 *Salvia* species. *Mentha longifolia*, *M. spicata* and *Glechoma longituba* were used as an outgroup. Bootstrap values with 1,000 replicates were shown under each branch.

## Data Availability

The chloroplast genome assembled in this study are available in GenBank database of National Center for Biotechnology Information (NCBI, https://www.ncbi.nlm.nih.gov) under the accession number MT156374. The associated BioProject, Bio-Sample and SRA numbers are PRJNA831680, SAMN27760654 and SRR18909752, respectively.
